# Pre-participation Evaluation in Recreational High-Intensity Sports: A Literature Review

**DOI:** 10.7759/cureus.88530

**Published:** 2025-07-22

**Authors:** Smita Kanase, Anandh S.

**Affiliations:** 1 Sports Physiotherapy, Krishna Vishwa Vidyapeeth (Deemed to be University), Karad, IND; 2 Community Health Physiotherapy, Krishna Vishwa Vidyapeeth (Deemed to be University), Karad, IND

**Keywords:** cardiovascular fitness (cvf), high-intensity sports, medical history, physical fitness, pre-participation evaluation, recreational sports, relevant physical examination, sports-specific assessment

## Abstract

The purpose of this review is to examine the development, validity, and reliability of pre-participation evaluation (PPE) in recreational athletes engaged in high-intensity sports (HISs). Recreational athletes, who participate in sports for enjoyment, fitness, and social engagement, are increasingly involved in high-energy activities such as swimming, tennis, and badminton. PPE is essential for identifying potential health risks, assessing fitness levels, and ensuring that athletes are physically prepared for the demands of these sports. This review highlights various PPE components, including medical history, physical examination, sport-specific assessments, and psychological readiness, with an emphasis on minimizing injury risk and optimizing performance. It also addresses implementation challenges, such as lack of awareness, misinterpretation of results, and the need for sport-specific evaluations. While current PPE practices offer significant benefits, limitations such as reliance on static assessments and the need for more dynamic evaluations are noted. The review concludes that integrating advanced technologies, such as wearable devices and real-time data analytics, could enhance the effectiveness of PPE protocols and improve injury prevention in recreational HISs. Overall, the findings emphasize the need for personalized and tailored PPE procedures to better serve the diverse needs of recreational athletes.

## Introduction and background

In recent times, sports has become an essential component of human life, contributing significantly to physical fitness, strength, and overall well-being. Across all stages of life, participation in sports holds considerable importance. Sports encompass activities, games, or competitions that involve physical exertion and skill, conducted according to established rules for enjoyment and personal development [1]. Participation in sports plays a pivotal role in an individual's holistic growth by cultivating life skills and enhancing intellectual, moral, and spiritual development while also strengthening personal character [2]. These developmental benefits significantly contribute to improved athletic performance, particularly in high-intensity sports (HISs). HISs involve activities that require elevated physical effort, typically based on aerobic or anaerobic training. Such exercises greatly increase energy expenditure and calorie burn, thereby enhancing athletic performance. Examples of HISs include swimming, cycling, tennis, basketball, boxing, sprinting, and weightlifting. Many of these sports incorporate high-intensity interval training (HIIT), a widely recognized exercise method with substantial effects on the musculoskeletal system. While HIIT offers numerous physiological benefits, it also raises the risk of musculoskeletal injuries [3]. To mitigate such risks, pre-participation evaluation (PPE) serves as a vital, evidence-based strategy, especially for athletes involved in HISs. PPE aims to identify individuals at increased risk of injury, promoting safer participation by detecting pre-existing medical conditions that may compromise safety during physical exertion. This early detection enables timely intervention, reducing the likelihood of adverse outcomes, including severe injury or death [4].

Although sports participation is generally encouraged, many individuals opt to specialize in specific sports, such as football, basketball, baseball, or tennis, based on their interests and inherent talents [5]. This trend has led to a growing population of recreational athletes, individuals who regularly engage in physical activities without the same level of intensity or commitment as competitive athletes. Recreational players are often assigned to teams without specific consideration of skill level. Notably, recent studies indicate that 39.21% of recreational athletes have experienced injuries during or after gameplay. These injuries typically cause pain and stiffness, although they are rarely severe enough to require surgical intervention [6]. Implementing PPE among recreational athletes is essential to minimizing such risks. It helps prevent potential injuries and manage the long-term effects of physical exertion. The PPE process includes components such as medical and family history, physical examination, consideration of male and female athlete triads, assessments of heat tolerance and hydration status, anthropometric measurements, nutritional evaluations, and musculoskeletal screenings [7]. These elements are critical in evaluating the development, validity, and reliability of recreational athletes' physical capacities. The constructs of development, validity, and reliability are fundamental in assessing athletic skill and performance. These are commonly evaluated through structured fitness tests such as the T-run agility test and the beep test. Such assessments are crucial in sports analytics, ensuring performance metrics and probability estimates are both accurate and dependable. These tools are especially effective in measuring offensive contributions, where there is typically more consensus on player performance compared to defensive roles [8]. As a result, technical assessments based on validated and reliable methods support performance evaluation and contribute to individual athlete development. These evaluations are invaluable for coaches, enabling them to tailor training regimens, identify specific strengths and weaknesses, and make informed decisions about athlete progression [9]. Ultimately, the analysis of field-based performance metrics, through parameters such as test development, validity, reliability, and duration, serves to measure movement skill competency in lifelong physical activities [10].

## Review

Literature review

Recreational athletes participate in sports and physical activities primarily for enjoyment, fitness, and personal challenge, rather than with the intention of professional competition. These individuals are often highly enthusiastic about their chosen sports and are motivated to improve both their performance and overall well-being. Common HISs practiced by recreational athletes include football, tennis, and badminton. HISs involve activities that place significant physical demands on the body. The appropriate level of exercise intensity is typically determined by a combination of an individual's health status, fitness goals, and current physical condition. Exercise intensity is traditionally classified as low, moderate, or vigorous, with HISs falling into the vigorous category, requiring substantial cardiovascular and musculoskeletal engagement.

In such sports, evaluating the validity and reliability of pre-participation screening is especially important. PPE serves as a practical and systematic approach for assessing an individual's readiness to participate and for identifying potential health risks. It plays a crucial role in establishing validity (how accurately the assessment measures what it is intended to measure) and reliability (the consistency and repeatability of results) in performance-related data.

This review provides an overview of the importance of PPE in HISs, particularly among recreational athletes. It examines how PPE can be used to assess various health and performance parameters relevant to HISs. Additionally, it highlights key components of PPE, such as neuromuscular assessments, psychological readiness, and mental health screenings, all of which are essential for ensuring safe and effective participation in high-intensity physical activities.

Overview of HISs

HISs provide a valuable platform for assessing the body's physiological responses, particularly in relation to muscular stress, elevated systemic oxidative levels, and associated molecular signaling pathways [11]. Participation in HISs has been shown to elicit significant physiological adaptations, especially among untrained individuals. For example, HIIT, a common component of HISs, has been linked to substantial improvements in maximal oxygen uptake (VO₂ max), a key indicator of cardiovascular and aerobic fitness [12]. HIIT-based training, often employed in both competitive matches and structured practice sessions, facilitates a deeper understanding of the relationship between training intensity, match demands, and overall fitness levels [13]. These fitness attributes are essential for designing effective training stimuli aimed at enhancing aerobic capacity and optimizing performance in HISs [14]. Representative HISs discussed in this review include swimming, cycling, soccer, tennis, basketball, field hockey, and boxing. Although existing research on the application and outcomes of HISs in recreational settings remains limited, this review expands the current body of literature by incorporating a broader range of sport-specific examples to enhance the understanding of HIS-related evaluation methods and outcomes.

PPE of recreational players in HISs

PPE serves as a vital screening tool designed to assess health risks, particularly among sedentary individuals who intend to begin a structured fitness program or participate in high-intensity physical activities [15]. PPE plays a preventive role by identifying underlying medical conditions or risk factors that may predispose individuals to injury or adverse health events during physical exertion. In the context of HISs, the PPE process considers several critical factors, including the following: relative contribution of age, stage of puberty, anthropometric characteristics, health-related physical fitness, sport-specific performance tests, and match-related technical proficiency [16].

These parameters provide a holistic understanding of the individual's physical readiness and inform training or medical decisions. Physical activity, as assessed in PPE, is not only essential for performance but also plays a foundational role in addressing health concerns such as low muscle mass, metabolic syndrome, and other chronic conditions [17]. Recreational players, distinct from professional athletes, engage in sports and physical activities for enjoyment, general well-being, and stress relief rather than competition or career advancement. Examples include joggers, runners, dancers, as well as participants in football, basketball, volleyball, and martial arts. These individuals often train without the intensity, frequency, or resources typical of professional athletes, making PPE a particularly valuable tool for identifying risks and guiding safe participation in HISs.

The risk of sports-related injuries remains notably high among athletes, including recreational players. Ensuring that individuals are both physically and psychologically prepared is essential for a safe return to sports participation [18]. Physical activity inherently carries a risk of injury, with some, such as anterior cruciate ligament (ACL) ruptures, being particularly severe and commonly associated with HIS participation [19].

Within the PPE framework, component analysis aims to assess an athlete's current physical and psychological condition to optimize performance while minimizing health risks [20]. By evaluating factors such as exercise intensity, workload volume, and energy expenditure, PPE enables healthcare professionals to draw informed comparisons across different forms of physical activity, particularly from the perspective of health and performance optimization [21].

A comprehensive PPE for recreational athletes engaged in HISs typically includes the following core components: physiological assessments (evaluation of muscular strength, flexibility, cardiovascular endurance, and joint stability), neuromuscular evaluations (assessment of balance, coordination, and motor control, which are critical for efficient movement and injury prevention), and psychological readiness and mental health screening (examination of mental preparedness, motivation, stress levels, and potential risk of psychological burnout).

The importance of these PPE components is supported by a growing body of research. For instance, flexibility has been identified as a key factor in safe performance. Studies by Sylejmani et al. and Jackson underscore the value of flexibility assessments in determining recreational athletes' readiness for participation [22,23]. Gomez-Espejo et al. emphasize psychological readiness as a critical determinant in return-to-play decisions and in reducing the risk of injury recurrence [24]. Similarly, Sannicandro et al. highlight the relevance of neuromuscular assessments, such as the one-leg hop test, for evaluating movement quality and control in soccer players [25]. In endurance sports, a 2019 study on recreational marathon runners recommends including lower extremity strength assessments to accurately measure fitness and prevent overuse injuries [26]. Together, these components offer a multidimensional evaluation of the athlete's condition, ensuring they are physically and mentally prepared to safely engage in HISs. A well-structured PPE not only helps prevent injuries but also informs training modifications and supports comprehensive athletic development.

Validity and reliability of PPE for recreational players in HISs

In the context of PPE, two critical parameters that determine the effectiveness of assessment tools are validity and reliability. Validity refers to the extent to which a test accurately measures the key components of the task or concept it is designed to assess [27]. In HISs, this means ensuring that evaluation tools genuinely reflect the physiological, neuromuscular, and psychological demands of the sport. Reliability, on the other hand, refers to the consistency and repeatability of test results under similar conditions over time.

Several studies have explored these psychometric properties in relation to PPE components for recreational athletes. For example, a study evaluating a novel agility and skill test for female amateur soccer players found the test to be both valid and reliable for assessing integrative movement skills and agility [28]. Researchers used paired t-tests and correlation analyses to assess test-retest reliability and overall utility. The test demonstrated strong reliability indicators, supporting its application in performance evaluation. However, a noted limitation was the constrained spatial setup of the testing environment, which may impact the generalizability of the results.

Another relevant study examined the validity of various jump tests in professional cerebral palsy football players using a cross-sectional design [29]. The research assessed the tests' ability to accurately measure jump height and lower-body power. The findings demonstrated high levels of both validity and reliability, indicating that such assessments can be effectively applied, even in specialized athlete populations, to evaluate functional performance.

Together, these studies underscore the importance of selecting and validating assessment tools tailored to the physical and psychological profiles of recreational athletes engaged in HISs. A valid and reliable PPE ensures that decisions regarding player readiness, training modifications, or return-to-play are evidence-based, objective, and standardized. In conclusion, incorporating scientifically validated and reliable tests into PPE protocols enhances both safety and performance outcomes for recreational athletes while providing sports scientists, coaches, and clinicians with accurate tools for long-term athletic monitoring and development.

Challenges and limitations of PPE in recreational HISs

PPE plays a vital role in assessing an individual's health and fitness prior to engaging in physically demanding activities, especially in HISs. It helps identify potential health risks and ensures that recreational athletes are fit for participation. However, despite its recognized importance, several challenges and limitations hinder the effective implementation of PPE in recreational HIS settings. These challenges often stem from the informal nature of recreational sports and the wide variability in participants' commitment levels, physical fitness, and understanding of personal health. PPE in recreational HISs is a critical step in assessing an individual's health and fitness before engaging in physically demanding activities [30]. However, there are several challenges and limitations associated with PPE in these settings [31], particularly due to the nature of recreational sports and the varying levels of participant commitment, fitness, and understanding of personal health. Some of the key challenges are explained in this section.

A study by Butler and Dzikus on the leisure activities of American professional basketball players highlighted the influence of sports labor migration on recreational behavior and emphasized the need for greater PPE awareness. However, a key limitation was the limited availability of educational resources, which may have affected players' understanding and adoption of PPE protocols [32].

Similarly, research examining performance metrics in basketball, such as defensive rebounds and assists, found correlations with match outcomes but faced data interruption issues, which compromised the robustness of findings [33]. This reflects a broader challenge in sports research, namely, inconsistent data collection, which hinders the validation and practical application of PPE tools.

Another study focusing on injury prevention among basketball coaches and players found that, although preventive strategies were generally implemented, athletes often engaged in insufficient warm-up and preparation activities before games. Moreover, there was a lack of proactive follow-up on medical advice, undermining the long-term benefits of PPE [34].

In a Lebanese basketball context, a cross-sectional analysis revealed significant gaps in knowledge and perception regarding PPE. Despite high response rates from both athletes (97%) and coaches (69%), the study found that awareness of PPE and injury prevention remained low, representing a common barrier in recreational sports where athletes may not prioritize structured evaluations [35].

These findings point to several recurring challenges in PPE for recreational HISs. Recreational athletes, unlike their competitive counterparts, rarely undergo systematic training or medical assessments, leaving them less equipped to understand or manage injury risks. This issue is compounded by the perception that recreational sports are inherently less demanding, even though injuries in HISs are prevalent.

Recent data trends from 2021 to 2023 show a rising incidence of recreational injuries in HISs, with reported increases of 20%, 12%, and 2% over the three years. This rise corresponds with the growing popularity of high-intensity activities, such as HIIT workouts, which combine maximal effort with variable recovery times. In such contexts, PPE becomes crucial for minimizing injury risk and ensuring safe, sustainable participation.

Physical limitations also pose a challenge in PPE implementation. Pre-existing injuries, chronic conditions, disabilities, or age-related factors can affect performance and increase vulnerability to injury. Identifying such limitations through PPE is essential for tailoring training programs to the individual's capabilities, thereby reducing injury risk and supporting overall athletic development.

The limitations of PPE in recreational HISs largely arise from a lack of awareness, insufficient education, inconsistent data collection, and diverse physical capabilities among athletes. Nevertheless, PPE remains a critical tool for promoting safety and enhancing performance in high-intensity recreational sports. Continued efforts are needed to improve access to educational resources, standardize evaluation methods, and raise awareness among recreational athletes and coaches to maximize the effectiveness of PPE protocols.

Key debates

There has been considerable debate surrounding the establishment, validity, and reliability of PPE for recreational athletes engaged in HISs. One major point of contention is whether PPE should be standardized across all sports or customized to the specific physical demands of each sport. Advocates for customization argue that sport-specific evaluations better account for an athlete's prior performance, fitness level, and injury history, while proponents of standardization emphasize the importance of a universal framework for consistency and comparability. Another key issue concerns the validity of current PPE tests; while useful for detecting general health risks like cardiovascular or musculoskeletal conditions, these tests are often criticized for their limited ability to predict injuries specific to HISs, particularly those caused by fatigue, overuse, or high-intensity stress. Additionally, the reliability of PPE, referring to the consistency of results over time and between evaluators, has been questioned due to factors such as inconsistent test administration, subjective interpretation of results, and external influences like environmental conditions or an athlete's mood. These concerns raise doubts about the robustness of PPE as a predictive tool for injury prevention or physical readiness. Despite broad consensus on the value of PPE in enhancing safety and performance, the challenge remains in developing effective, ethical, and practical guidelines that address the diverse needs of recreational athletes while maintaining the relevance and efficacy of PPE protocols.

Practical implications

The examination of the design, validity, and reliability of PPE in recreational athletes involved in HISs has significant practical implications for both future research and real-world applications. As HISs continue to rise in popularity, the need for effective and reliable PPE protocols becomes increasingly important. One key implication is the need to customize PPE procedures to address the specific physical demands and injury risks of different HISs, taking into account the unique characteristics of each sport and the varying fitness levels of recreational athletes. Additionally, integrating wearable technologies and mobile health applications, such as Global Positioning System (GPS) trackers and heart rate monitors, can enhance PPE by enabling real-time data collection and providing dynamic, individualized assessments. There is also a pressing need to validate PPE protocols for their ability to predict injury risk and assess performance outcomes in high-intensity environments, moving beyond basic health screenings to offer actionable insights for injury prevention and athletic improvement. Furthermore, incorporating psychological readiness into PPE is essential, as mental well-being, stress management, and focus are critical to safe and effective sports participation. Cost-effectiveness and accessibility remain major challenges, especially for recreational athletes; therefore, future efforts should aim to develop affordable, scalable PPE solutions, such as community-based screenings and low-cost digital tools. Together, these implications underscore the need for tailored, technology-enhanced, psychologically informed, and accessible PPE protocols to improve safety, performance, and participation in HISs among recreational athletes.

## Conclusions

PPE is vital for ensuring the safety and preparedness of recreational athletes engaged in HISs such as tennis, swimming, and badminton. Although these athletes participate for enjoyment and fitness rather than competition, they face significant physical demands. PPE assesses medical history, physical condition, and sport-specific factors, accounting for gender and individual differences, to evaluate suitability for participation and reduce injury risks. Training for HISs enhances strength, power, flexibility, and performance, and PPE supports this by identifying potential health concerns. However, challenges such as limited awareness, false security, and communication gaps hinder PPE effectiveness. The future of PPE is promising, with technologies like wearables and real-time data offering more personalized, accurate, and dynamic assessments. Continued innovation and improved awareness are key to maximizing the benefits of PPE for recreational athletes.
